# Assessment of exercise-induced stress by automated measurement of salivary cortisol concentrations within the circadian rhythm in Japanese female long-distance runners

**DOI:** 10.1186/s40798-020-00269-4

**Published:** 2020-08-24

**Authors:** Kazumi Ushiki, Katsuhiko Tsunekawa, Yoshifumi Shoho, Larasati Martha, Hirotaka Ishigaki, Ryutaro Matsumoto, Yoshimaro Yanagawa, Asuka Nakazawa, Akihiro Yoshida, Kiyomi Nakajima, Osamu Araki, Takao Kimura, Masami Murakami

**Affiliations:** 1 Department of Clinical Laboratory Medicine, Gunma University Graduate School of Medicine, 3-39-22 Showa-machi Maebashi, Gunma, 371-8511 Japan; 2 Clinical Laboratory Center, Gunma University Hospital, 3-39-15 Showa-machi Maebashi, Gunma, 371-8511 Japan; 3 Faculty of Education, Ikuei University, 1-7-1 Tonya-machi Takasaki, Gunma, 370-0006 Japan; 4 Department of Medical Technology, Faculty of Health Science, Gunma Paz University, 1-7-1 Tonya-machi Takasaki, Gunma, 370-0006 Japan

**Keywords:** Sequential saliva sampling, Exercise stress, Overtraining syndrome, Electrochemiluminescence immunoassay (ECLIA)

## Abstract

**Background:**

Overtraining syndrome, caused by prolonged excessive stress, results in reduced performance and cortisol responsiveness in athletes. It is necessary to collect saliva samples sequentially within circadian rhythm for assessing exercise stress by measuring cortisol concentrations, and automated cortisol measurements using electrochemiluminescence immunoassay (ECLIA) may be useful for measuring a large number of saliva samples. In this study, we evaluated the appropriate use of cortisol-based exercise stress assessment within the circadian rhythm, which may diagnose and prevent overtraining syndrome in athletes.

**Methods:**

We collected saliva and sera from 54 healthy participants and analyzed the correlation between salivary cortisol concentrations measured by ECLIA and enzyme-linked immunosorbent assay (ELISA) or serum cortisol analysis. We also collected saliva continuously from 12 female long-distance runners on 2 consecutive days involving different intensities and types of exercise early in the morning and in the afternoon and measured salivary cortisol concentrations using ECLIA. Each exercise intensity of runners was measured by running velocities, Borg Scale score, and rate of change in the pulse rate by exercise.

**Results:**

ECLIA-based salivary cortisol concentrations correlated positively with those detected by ELISA (*ρ* = 0.924, *p* < 0.001) and serum cortisol (*ρ* = 0.591, *p* = 0.001). In long-distance runners, circadian rhythm of salivary cortisol, including the peak after waking and the decrease promptly thereafter, were detected on both days by continuous saliva sampling. The rates of change in salivary cortisol concentrations were significantly lower after an early morning exercise than after an afternoon exercise on both days (day 1, *p* = 0.002, and day 2, *p* = 0.003). In the early morning exercise, the rate of change in salivary cortisol concentration was significantly higher on day 1 than on day 2 (*p* = 0.034), similar to a significant difference in running velocities (*p* = 0.001).

**Conclusions:**

Our results suggest that automated ECLIA-based salivary cortisol measurements are able to detect the athletes’ circadian rhythm and compare the exercise stress intensities at the same times on different days, even in the early morning, possibly leading to the prevention of overtraining syndrome.

## Key points

- Saliva samples should be collected sequentially within the circadian rhythm to assess the exercise-induced stress response by measuring the salivary cortisol concentration in athletes.
- The difference in the rate of change in salivary cortisol concentrations resulting from different exercise intensities could be compared at the same time on different days.
- Automated ECLIA methods that can measure cortisol concentrations in a large number of saliva samples may be useful for the diagnosis and prevention of overtraining syndrome.

## Background

Cortisol is a principal glucocorticoid that plays a crucial role in human metabolism and immune function. Cortisol secretion occurs according to the circadian rhythm and is regulated by the hypothalamus-pituitary-adrenal (HPA) axis [1]. Physical exercise, a physiological and psychological stressor, activates the HPA axis, thus increasing the secretion of cortisol in the adrenal cortex [2]. Athletes participating in various sports are often required to undertake excessive training workloads aiming at improving their performances. Although cortisol levels increase in proportion to exercise intensity and severity of environments in athletes [3], prolonged excessive stress reduces the cortisol response to exercise, resulting in a disorder named overtraining syndrome [4–6]. Overtraining syndrome also has been reported to result in the alteration of circadian rhythm, such as low level of resting cortisol [4] and peak loss at 30 min after awakening [7]. Because overtraining syndrome results in reduced athlete performance, assessment of circadian rhythms and responsiveness to exercise in cortisol secretion may be useful for diagnosis and prevention of overtraining syndrome.

Several studies have attempted to measure serum and salivary cortisol concentrations to evaluate stress resulting from various types and intensities of exercise [8–10]. Although serum specimens are often used in studies conducted under clinical and research settings, blood sampling needs to be performed by medical professional staff. Additionally, this procedure enhances cortisol secretion via venipuncture stress [11]. Salivary cortisol has several advantages with respect to exercise stress assessment, particularly a simple collection method that involves a stress-free, non-invasive procedure that does not require medical professional staff [10, 12]. Furthermore, salivary cortisol also has the characteristics of unconjugated cortisol and independent of salivary flow rate by free diffusion through the salivary gland, while serum cortisol is a steroid conjugated to corticosteroid-binding globulin (CBG) [13].

Conventionally, salivary cortisol concentrations have been determined manually using enzyme-linked immunosorbent assays (ELISAs). However, automated electrochemiluminescence immunoassays (ECLIAs) developed for measurement of serum cortisol concentration have been recently applied to saliva samples. The ECLIA-determined salivary cortisol concentrations were shown to be useful for the diagnosis of Cushing’s syndrome and adrenal insufficiency [14, 15]. Furthermore, salivary cortisol concentrations measured by second-generation ECLIAs showed a better positive correlation with those measured by liquid chromatography-tandem mass spectrometry (LC-MS/MS) than those measured by first-generation ECLIAs [16]. However, these automated methods have not been used to determine salivary cortisol concentrations in non-clinical settings, such as exercise training.

Notably, one previous study used ECLIA to establish reference intervals for the salivary cortisol circadian rhythm in healthy adults [16]. Physical exercise was previously shown to affect the circadian rhythm of cortisol [17–24], and many studies have determined serum and salivary cortisol concentrations as measures of exercise stress at various time points [10]. Changes in serum cortisol concentrations by exercise were reported to be greater in the evening than in the morning due to the circadian rhythm of cortisol secretion [25]. Diagnosis of overtraining syndrome in athletes may be possible to detect the adrenocortical dysfunction including the loss of circadian rhythm and the decrease in the response to exercise by salivary cortisol measurements [6, 7]. In addition, if the alterations in stress response due to different exercise intensities can be evaluated by salivary cortisol concentration, it may contribute to the prevention of overtraining syndrome. However, no definite criteria have been established regarding the ideal timing of assessments of different exercise-induced stresses based on salivary cortisol concentration within the circadian rhythm. To overcome this problem, it is important to monitor the salivary cortisol concentrations sequentially within the circadian rhythm, and this monitor requires continuous saliva collection throughout the days with exercise trainings and measurement of a large number of saliva samples. Automated cortisol measurement methods, such as ECLIA, may be useful to enable the continuous monitoring of salivary concentrations.

In the present study, we evaluated and compared the accuracy of salivary and serum cortisol concentrations measured automatically using second-generation ECLIA and salivary concentrations measured using a conventional highly sensitive ELISA method. We also collected saliva samples during two consecutive days with various types of exercise training in Japanese female long-distance runners and measured salivary cortisol concentrations by automated ECLIA methods. Thus, we verified the hypothesis that salivary cortisol is able to detect the stress responses induced by different intensities and types of exercise between different days even within the circadian rhythm.

## Methods

### Participants

This prospective study was conducted according to the Declaration of Helsinki, and the protocol was approved by the ethics committee of Gunma University Graduate School of Medicine (approval number 13-36, 14-89). All study subjects provided written informed consent prior to participation in the study.

Fifty-four young healthy Japanese participants (male 17; female 37) were enrolled in this study for correlation analyses of the accuracy of ECLIA-based salivary cortisol concentrations, with no exclusion criteria. Saliva samples were collected from all participants, while blood samples were collected from 27 participants (male 17; female 10). All samples were collected between 09:00 and 10:00.

We additionally enrolled 12 Japanese elite female runners who participated in the national women’s long-distance relay race in an analysis of the response to exercise stress within the circadian rhythm, with no exclusion criteria. All runners lived in the same dormitory, and their conditions, such as wakeup time, mealtime, bedtime, and meal content, were standardized. We collected salivary samples sequentially from these runners on 2 consecutive days involving different intensities and types of training in the early morning and afternoon on the schedule away from the race. The runners were subjected to the following exercise program: day 1, 10,000-m fixed-distance speed running in the early morning and interval training with 4 sets of 1200-m fast running and 800-m light jogging in the afternoon; day 2, fixed-speed running for 50 min in the early morning and afternoon. The runners drank enough water to avoid dehydration during these trainings. On both days, saliva samples were collected at 8 time points: upon waking (05:00), before early morning exercise (06:00), after an early morning exercise (07:00), before breakfast (08:00), before lunch (12:00), before afternoon exercise (14:00), after afternoon exercise (16:00), and before dinner (19:00).

### Physical examinations

Participants’ height and weight were measured, and the body mass index was calculated as the weight divided by the squared height (kg/m^2^). Information about the use of medications was obtained through interviews. For the female runners, the distance and duration of running during each training session were measured, and the running velocity was calculated as the distance divided by the duration (m/min). The Borg Scale [26] was used to determine the rating of perceived exertion (RPE) of the runners after exercise. Runners’ pulse rates were measured before and after exercise, and the rate of change in the pulse by exercise was calculated as the pulse rate after exercise divided by the pulse rate before exercise (%).

### Sample collection

Saliva samples were collected using Salivette® cotton swabs (Sarstedt, Nümbrecht, Germany) as previously described [14, 16]. In brief, the subjects did not consume any food or drink except water within 15 min before sample collection. All saliva samples were centrifuged (1500×*g*) at 4 °C for 10 min and subsequently stored at − 20 °C until analysis. Blood samples were obtained by puncturing an antecubital vein using a 23-G needle while the subject was in a seated position. Serum samples were separated from blood by centrifugation (1500×*g*) at 4 °C for 10 min and stored at − 80 °C until analysis.

### Measurements of salivary and serum cortisol concentrations

ECLIA measurements of salivary and serum cortisol concentrations were performed using the Elecsys Cortisol II on Modular Analytics E170 system (Roche Diagnostics K.K, Tokyo, Japan) [16, 27]. Intra- and inter-assay coefficients of variation were 4.1% and 4.3% for saliva and 0.7% and 1.5% for serum, respectively. ELISA measurements of salivary cortisol concentrations were performed using a kit (cortisol EIA kit, Expanded Range, High Sensitivity, Salivary; Salimetrics LLC, Carlsbad, CA, USA) according to the manufacturer’s instructions [28, 29]. The rate of change in the salivary cortisol concentration by exercise in female runners was calculated as the salivary cortisol concentration after exercise divided by the salivary cortisol concentration before exercise (%).

### Statistical analysis

The results of each measurement are expressed as median values and corresponding 25th–75th percentile ranges. Spearman’s correlation analyses were used to assess the correlations of the salivary cortisol concentrations determined by ECLIA or ELISA and the serum cortisol concentrations determined by ECLIA. The Wilcoxon signed-rank test was used to identify statistically significant differences in running velocities, Borg Scale scores, rates of change in the pulse rate, salivary cortisol concentrations, and rates of change in the salivary cortisol concentrations between 2 different time points. A *p* value of < 0.05 was considered to indicate a significant difference, and each exact *p* value was described in the “Results” section. All statistical analyses were performed using SPSS Statistics, version 25.0 (SPSS, Chicago, IL, USA).

## Results

### Correlations of salivary cortisol concentrations determined by ECLIA or ELISA and Serum cortisol concentrations

**Fig. 1:**
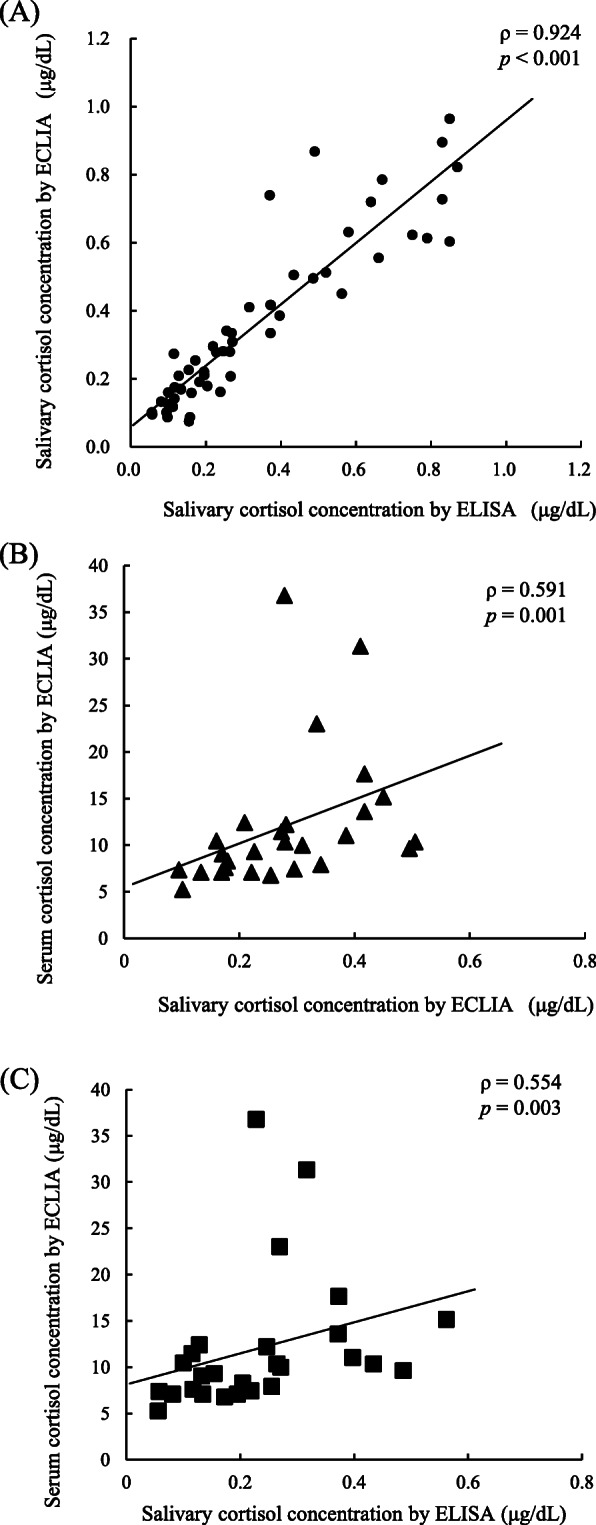
Correlations between the salivary cortisol concentrations measured by ECLIA and ELISA (**a**, *n* = 54), between the salivary and serum cortisol concentrations measured by ECLIA (**b**, *n* = 27), and between the salivary cortisol concentration measured by ELISA and serum cortisol concentration measured by ECLIA (**c**, *n* = 27) in healthy participants. ECLIA; electrochemiluminescence immunoassay, ELISA; enzyme-linked immunosorbent assay

Table 1 presents the characteristics of 54 young healthy Japanese participants included in this analysis of the accuracy of automated ECLIA-based salivary cortisol concentrations. The results of Spearman’s correlation analyses comparing the concentrations of salivary cortisol determined by ELISA and ECLIA in 54 participants and of serum cortisol in 27 participants are shown in Fig. 1. The salivary cortisol concentrations determined by ECLIA and ELISA exhibited a strong positive correlation (*ρ* = 0.924, *p* < 0.001). Reference interval which corresponds to 2.5th–97.5th percentile of salivary cortisol concentrations measured by ECLIA was 0.078–0.938 μg/dL between 09:00 and 10:00 in 54 healthy participants. This interval was almost equivalent to the reference interval for salivary cortisol concentrations by ECLIA at about the same time point in the report by Gagnon et al. [16]. Moreover, whereas the salivary cortisol concentrations determined by both methods correlated positively with the serum cortisol concentrations determined by ECLIA, a stronger correlation was observed with the ECLIA-based salivary cortisol concentrations (salivary cortisol by ECLIA and serum cortisol, *ρ* = 0.591, *p* = 0.001; salivary cortisol by ELISA and serum cortisol, *ρ* = 0.554, *p* = 0.003). There was no difference between male and female participants in each correlation result (data not shown).

**Table 1:** Characteristics of healthy participants included in the correlation analysis

	All (*n* = 54)	Male (*n* = 17)	Female (*n* = 37)
Age (years)	23.0 (20.0–24.3)	23.0 (23.0–26.5)	23.0 (20.0–24.0)
Height (cm)	162.7 (158.0–170.0)	175.0 (169.0–177.5)	159.7 (157.0–163.0)
Weight (kg)	54.8 (48.9–59.9)	62.5 (59.4–70.3)	52.0 (47.9–55.4)
Body mass index (kg/m^2^)	20.6 (18.9–21.6)	21.2 (19.9–22.1)	20.3 (20.3–21.4)

Data are expressed as medians (25th–75th percentiles)

### Running intensity of each exercise program performed by female long-distance runners

Table 2 shows the characteristics of 12 female long-distance runners who were included in an evaluation of the stress response to exercise. Two runners took oral iron medicine. One runner dropped out of the study during the afternoon exercise on day 2 because of poor condition. Table 3 depicts differences of the running intensities of the female runners during each exercise program. The running distance of interval training in the afternoon exercise on day 1 was 8000 m, which was shorter than the distances in other exercises. In addition, running velocity of interval training which included fast running with a maximum velocity of 400 m/min and slow jogging for recovery was calculated as the average velocities using the total training time and was compared with the velocities of other exercises. The running velocity was significantly higher in the early morning exercise on day 1 than in afternoon exercise on day 1 (*p* = 0.026). Moreover, the running velocities were significantly higher in the early morning exercise and the afternoon exercise on day 1 than on day 2, respectively (early morning exercise, *p* = 0.001, and afternoon exercise, *p* = 0.002). However, the running velocity did not differ significantly between the early morning exercise and afternoon exercise on day 2 (*p* = 0.317), and no significant differences were observed in the Borg Scale scores and the rates of change in the pulse rate.

**Table 2:** Characteristics of Japanese female long-distance runners

Characteristic	Value
Number	12
Age (years)	22.0 (19.3–24.8)
Height (cm)	160.0 (153.3–162.8)
Weight (kg)	45.5 (41.5–48.0)
Body mass index (kg/m^2^)	17.8 (16.7–18.5)
Number of runners taking oral iron medicine	2

Data are expressed as medians (25th–75th percentiles)

**Table 3:** Running intensities during each exercise program performed by female long-distance runners

	Day 1		Day 2		*p*_*1*_	*p*_*2*_	*p*_*3*_	*p*_*4*_
	Early morning exercise	Afternoon exercise	Early morning exercise	Afternoon exercise				
Exercise program	10,000-m fixed running	Interval training	50 min fixed-speed running	50 min fixed-speed running				
Running distance (m)	10,000	8000	10,000	10,000				
Running velocity (m/min)	238 (238–240)	222 (222–227)	200 (200–200)	200 (200–200)	0.026	0.317	0.001	0.002
Borg Scale score	12.0 (10.3–13.0)	13.0 (12.0–15.0)	13.0 (12.0–13.0)	13.0 (12.0–15.0)	0.081	0.380	0.230	1.000
Rate of change in the pulse rate (%)	185.0 (142.3–191.5)	166.0 (149.3–198.8)	164.5 (129.3–196.8)	147.0 (138.0–185.0)	0.754	0.286	0.307	0.213

Data are expressed as medians (25th–75th percentiles)

*p*_*1*_ early morning exercise vs. afternoon exercise on day 1, *p*_*2*_ early morning exercise vs. afternoon exercise on day 2, *p*_*3*_ day 1 vs. day 2 in early morning exercise, *p*_*4*_ day 1 vs. day 2 in afternoon exercise

### Responses of salivary cortisol concentrations to exercise during circadian rhythms on 2 consecutive days in female runners

**Fig. 2:**
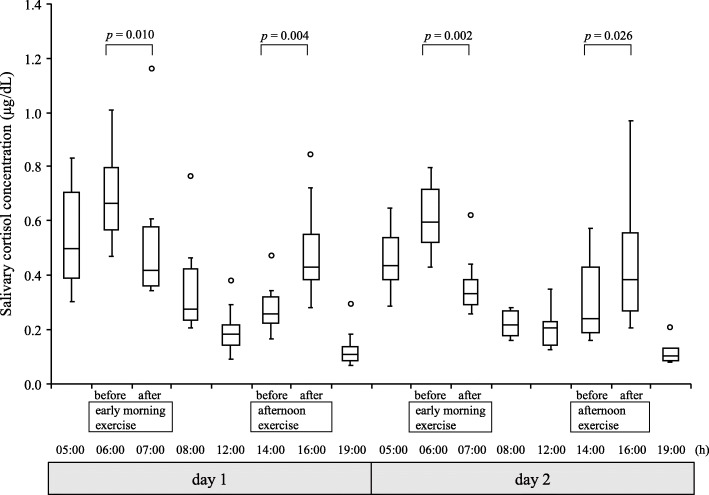
Changes in salivary cortisol concentration in response to each exercise program according to circadian rhythm on 2 consecutive days in female long-distance runners

Figure 2 demonstrates changes in the salivary cortisol concentrations in response to exercise within the circadian rhythms of the female runners over a period of 2 consecutive days. The salivary cortisol concentrations peaked after waking (06:00) and decreased promptly thereafter on both days. Moreover, the salivary cortisol concentrations decreased significantly after an early morning exercise on both days but increased significantly after afternoon exercise on both days. Additionally, the salivary cortisol concentrations decreased to equally low levels in the evening on both days, despite the different types of exercise.

### Rate of change in salivary cortisol concentrations resulting from exercise in female runners

**Fig. 3:**
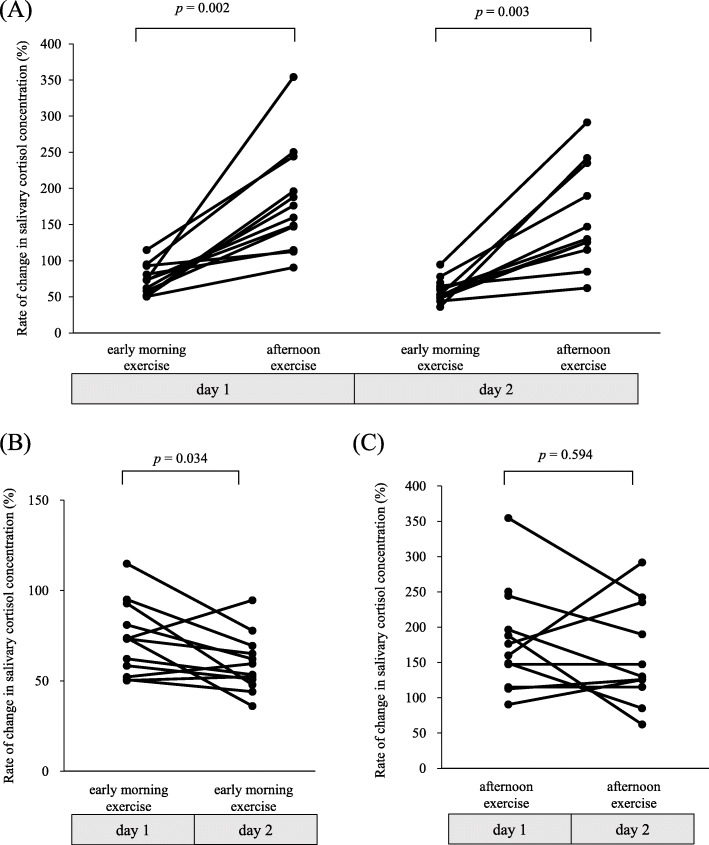
Comparison of the rate of change in the salivary cortisol concentration resulting from exercise between the early morning and afternoon time points on days 1and 2 (**a**), between the early morning time points on days 1 and 2 (**b**), and between the afternoon time points on days 1 and 2 (**c**) in female long-distance runners

Figure 3 depicts comparisons in the rates of change in salivary cortisol concentrations resulting from exercise between 2 different time points in female runners. On both days, the rates of change in the salivary cortisol concentration were significantly lower in the early morning exercise than in the afternoon exercise (day 1, *p* = 0.002, and day 2, *p* = 0.003; Fig. 3a). In the early morning exercise, the rate of change in the salivary cortisol concentration was significantly higher on day 1 than on day 2 (*p* = 0.034; Fig. 3b). In contrast, there was no significant difference in the rates of change in the salivary cortisol concentrations resulting from the afternoon exercise on days 1 and 2 (*p* = 0.594; Fig. 3c).

## Discussion

In this study, we have demonstrated that automated ECLIA measurements of salivary cortisol levels can be used to accurately assess the stress responses to several intensities and types of exercise within the circadian rhythm. First, we observed positive correlations of the salivary cortisol concentration measured by ECLIA with that measured by ELISA and the serum cortisol concentration measured by ECLIA in healthy participants. Second, in an analysis of female long-distance runners, we could detect the presence of circadian rhythm of cortisol in saliva collected at 16 points during 2 days using an automated ECLIA method and observed a significantly lower rate of change in the salivary cortisol concentration following exercise in the early morning compared to the one in the afternoon. Additionally, we observed significant differences in the rates of change in cortisol concentrations resulting from early morning exercise programs with different intensities on two different days.

Previous studies have reported the accuracy of automated measurements of salivary cortisol concentrations. For example, a study of healthy subjects revealed a positive correlation between the salivary cortisol concentrations determined by first-generation ECLIA and by LC-MS/MS. However, the same study found that ECLIA yielded significantly higher salivary cortisol concentrations when compared to LC-MS/MS in patients with adrenocortical carcinoma who were treated with mitotane [30]. Another study of healthy individuals observed a positive correlation between the salivary cortisol concentrations determined by first-generation ECLIA and by high-sensitivity ELISA [28]. Second-generation ECLIA was superior to first-generation ECLIA in terms of yielding a salivary cortisol concentration that correlated positively with the concentration determined by LC-MS/MS [16]. Consistent with those studies, we observed a positive correlation between the salivary cortisol concentrations determined using second-generation ECLIA and high-sensitivity ELISA. Conversely, however, a previous study reported a moderate linear correlation between the salivary cortisol concentration determined using an enzyme-based immunoassay and the serum cortisol concentration determined using a radioimmunoassay [29]. In that study, the association between the serum and saliva cortisol concentrations was stronger when the data were fitted to an exponential model, indicating the salivary cortisol was not affected by CBG [29]. Consistent with that study, we also observed a moderate correlation between the salivary and serum cortisol concentrations. Furthermore, there was no difference between men and women in these correlation results of healthy participants, which was in agreement with the previous studies [16, 28–30]. Because the concentration of the salivary cortisol is reportedly independent of the salivary flow rate [10, 13, 18], we assessed the exercise-induced stress by salivary cortisol concentrations without considering the change of the salivary flow rate and viscosity caused by dehydration or sweating in female runners. Thus, we could use a large number of saliva specimens with the advantage of sampling and use the automated ECLIA method with the advantage of being able to measure salivary cortisol concentrations accurately and easily in this study. Further studies may reveal the usefulness of salivary cortisol concentration measurement for exercise-stress assessment by comparing with salivary flow rate, viscosity, and several indicators including enzymes, hormones, and bioactive substances studied in the previous report [31].

Previous studies reported the effects of physical exercise that could lead to the risk of overtraining syndrome, on the circadian rhythms of cortisol secretion in healthy volunteers and various types of athletes [17–24]. One study found that the cortisol concentrations in response to exercise peaked at 07:00, whereas the maximal increase in the cortisol concentrations relative to the control condition was observed at 24:00 [17]. Data obtained in healthy young men suggest that exercise should be performed in the evening, which is associated with a more rapid recovery in the serum cortisol concentration [21]. This preference for evening exercise is further supported by the effects of immunosuppression in the early morning, as indicated by a decrease in salivary IgA levels and an increase in salivary cortisol levels in well-trained swimmers [18]. Resistance training with muscle damage altered the diurnal variations of serum creatine kinase and cortisol even after 48 h in well-trained men [23]. Moreover, the strenuous levels of training and competition faced by elite artistic gymnasts were found to eliminate the diurnal rhythm of salivary cortisol [20]. In contrast, swimming competition with high mood disturbances increased salivary cortisol levels after contests in the afternoon compared to control day, but did not alter the cortisol awakening response and the diurnal rhythms in male professional swimmers [24]. In the present study, the female long-distance runners’ salivary cortisol concentrations throughout the morning were within the reference interval for 54 healthy participants in this study. The runners’ salivary cortisol levels were also within the reference intervals at each different time point as defined previously [16], except after the afternoon exercise in both days. Moreover, the runners retained the circadian rhythm of salivary cortisol as indicated by a peak after waking and a return to a consistent level in the evening of both days, suggesting that they were not to suffer from overtraining syndrome. This observation was attributed to the performance of regular training without extreme physical and mental stress while not preparing for a race. In addition, our results indicate that it may be difficult to observe the response of salivary cortisol to exercise in the early morning compared to that in the afternoon because of the circadian rhythm, consistent with the previous study using serum cortisol concentrations [25]. Nevertheless, the significant difference in the rate of change in the early morning salivary cortisol concentrations on days 1 and 2 suggest that it may be possible to use salivary cortisol to compare the stress responses induced by different exercise intensities on different days at the same time, even in the early morning.

With respect to the exercise intensity, increased serum cortisol concentrations were observed in moderate- to high-intensity exercise, but not in low-intensity exercise, 40% of their maximal oxygen consumption (VO_2 max_) [19]. Although we did not measure the VO_2 max_ in our study, we used the running velocities, Borg Scale scores, and rates of change in the pulse rate to determine the exercise intensities. We attributed the lack of a significant difference in the rates of change in the pulse rate between 2 time points at least partly to the fact that the pulse rates measured at the end of the exercise program did not assess as maximum pulse rate during exercise. Furthermore, the lack of a significant difference in the Borg Scale scores is likely because this scale is used as a subjective measure of RPE and tends to be affected by individual differences. However, we observed a significant difference in the running velocities between different time points. This was the most objective indicator in our study, and it is noteworthy that the early morning running velocity was significantly higher on day 1 than on day 2. The higher rate of change in the early morning salivary cortisol concentration on day 1 thus presumably reflects the running velocity. In contrast, we did not observe a significant difference in the rates of change in the afternoon salivary cortisol concentrations between days 1 and 2, although the running velocities differed significantly at these time points. This observation may be due to differences in the types of exercise performed on the different days; interval training included a slower jogging for recovery and a shorter distance despite the faster maximum velocity, on day 1, and fixed-speed running, on day 2.

One of the limitations of this study is the relatively small sample size for the athletes to analyze stress response. We focused on enrolling the elite female long-distance runners living in a standardized environment as much as possible under the same conditions in regular training without extreme stress in a non-race season. Because the response of cortisol to exercise is greater in women than in men [32], further investigations are needed on male athletes. Another limitation is that we compared the responses of salivary cortisol concentrations between exercises of the same type at different intensities in the early morning and between exercises of different types in the afternoon on 2 consecutive days. However, we did not evaluate the exercise intensities using an accurate measure such as VO_2 max_. Future studies should compare the responses of salivary cortisol concentrations to multiple combinations of exercise types and intensities using more accurate indicators. We expect that further studies on stress markers in various types of athletes who require not only endurance strength but also instantaneous power will validate our hypothesis.

## Conclusions

The circadian rhythm identified by measuring salivary cortisol concentrations, which is effective in diagnosing overtraining syndrome, makes it difficult to evaluate differences in exercise-related stress throughout the day, and especially in the early morning. Nevertheless, it may be possible to overcome the effects of the circadian rhythm with the usage of salivary cortisol concentrations to assess the stress responses induced by different intensities of exercise at the same time on different days. This information suggests that the assessment of exercise-induced stress based on automated measurement of cortisol concentration in saliva collected sequentially within circadian rhythm may be useful not only for diagnosis but also for prevention of the overtraining syndrome in athletes. The present study suggests that sequential salivary cortisol measurement within circadian rhythm may contribute to providing each athlete with an appropriate training program.

## Data Availability

Please contact the corresponding author for reasonable data requests.

## Abbreviations

CBG: Corticosteroid-binding globulin
ECLIA: Electrochemiluminescence immunoassay
ELISA: Enzyme-linked immunosorbent assay
HPA: Hypothalamus-pituitary-adrenal
LC-MS/MS: Liquid chromatography-tandem mass spectrometry
RPE: Rating of perceived exertion
VO_2 max_: Maximal oxygen consumption
